# Large Regional Differences in Serological Follow-Up of Q Fever Patients in The Netherlands

**DOI:** 10.1371/journal.pone.0060707

**Published:** 2013-04-05

**Authors:** Gabriëlla Morroy, Cornelia C. H. Wielders, Mandy J. B. Kruisbergen, Wim van der Hoek, Jan H. Marcelis, Marjolijn C. A. Wegdam-Blans, Clementine J. Wijkmans, Peter M. Schneeberger

**Affiliations:** 1 Department of Infectious Disease Control, Municipal Health Service Hart voor Brabant, 's-Hertogenbosch, The Netherlands; 2 Department of Primary and Community Care, Academic Collaborative Centre AMPHI, Radboud University Nijmegen Medical Centre, Nijmegen, The Netherlands; 3 Department of Medical Microbiology and Infection Control, Jeroen Bosch Hospital, 's-Hertogenbosch, The Netherlands; 4 Centre for Infectious Disease Control, National Institute for Public Health and the Environment, Bilthoven, The Netherlands; 5 Laboratory of Medical Microbiology, St Elisabeth Hospital, Tilburg, The Netherlands; 6 Department of Medical Microbiology Laboratory for Pathology and Medical Microbiology (PAMM), Veldhoven, The Netherlands; University of Texas Medical Branch, United States of America

## Abstract

**Background:**

During the Dutch Q fever epidemic more than 4,000 Q fever cases were notified. This provided logistical challenges for the organisation of serological follow-up, which is considered mandatory for early detection of chronic infection. The aim of this study was to investigate the proportion of acute Q fever patients that received serological follow-up, and to identify regional differences in follow-up rates and contributing factors, such as knowledge of medical practitioners.

**Methods:**

Serological datasets of Q fever patients diagnosed between 2007 and 2009 (N = 3,198) were obtained from three Laboratories of Medical Microbiology (LMM) in the province of Noord-Brabant. One LMM offered an active follow-up service by approaching patients; the other two only tested on physician's request. The medical microbiologist in charge of each LMM was interviewed. In December 2011, 240 general practices and 112 medical specialists received questionnaires on their knowledge and practices regarding the serological follow-up of Q fever patients.

**Results:**

Ninety-five percent (2,226/2,346) of the Q fever patients diagnosed at the LMM with a follow-up service received at least one serological follow-up within 15 months of diagnosis. For those diagnosed at a LMM without this service, this was 25% (218/852) (OR 54, 95% CI 43–67). Although 80% (162/203) of all medical practitioners with Q fever patients reported informing patients of the importance of serological follow-up, 33% (67/203) never requested it.

**Conclusions:**

Regional differences in follow-up are substantial and range from 25% to 95%. In areas with a low follow-up rate the proportion of missed chronic Q fever is potentially higher than in areas with a high follow-up rate. Medical practitioners lack knowledge regarding the need, timing and implementation of serological follow-up, which contributes to patients receiving incorrect or no follow-up. Therefore, this information should be incorporated in national guidelines and patient information forms.

## Introduction

In the Netherlands, more than 4,000 patients were notified with acute Q fever during seasonal outbreaks between 2007 and 2010 [1], [2]. However, at least ten times as many people might have been infected with *Coxiella burnetii* in this period and had either asymptomatic or non-diagnosed infections [3], [4]. Acute Q fever may progress to chronic Q fever in about 2% of cases [5]. Chronic Q fever is not notifiable. There are no estimates for the proportion of asymptomatic acute *C. burnetii* infections that develop into chronic infection. The most common presentations of chronic Q fever are endocarditis and vascular infections, conditions with high morbidity and mortality [6]. The diagnosis of chronic Q fever is based on clinical presentation, presence of risk factors, diagnostic imaging techniques, detection of *C. burnetii* DNA in blood or tissue, and serological test results. Detection of an IgG antibody titre against phase I of *C. burnetii* of ≥1∶1,024 in a commercially available immunofluorescence assay during follow-up screening is considered an important marker of chronic infection [7]. Serological follow-up of acute Q fever patients is advised in order to identify and ensure timely treatment of chronic Q fever [8]–[10]. Follow-up is especially important for patients with valvulopathy, vascular prosthesis/abnormalities, pregnant women, and immunocompromised patients, as they have a higher risk of developing chronic Q fever after acute infection [8], [11].

A common but non-validated recommendation in the international literature was to offer all patients at least two serologic tests (at three and six months) in the first year after the diagnosis of acute Q fever [12], [13]. In 2008 the advice to test all Q fever patients at three, six, and twelve months after diagnosis was published in a Dutch microbiology journal [9]. Two years later, in 2010, new advice was published in another Dutch medical journal proposing one follow-up serologic test at nine months for low-risk patients, while the recommendation for high-risk patients was to test at three, six, nine, and twelve months [14]. During the Dutch Q fever outbreak, apart from these recommendations in scientific journal articles, there were no national guidelines on the serological follow-up of Q fever patients.

In the province of Noord-Brabant, one Laboratory of Medical Microbiology (LMM) used an automatic patient recall system for the serological follow-up of patients with acute Q fever. The other two LMMs depended on medical practitioners to request serological Q fever follow-up. The Municipal Health Service (MHS) Hart voor Brabant received information from both patients and health professionals that indicated poor serological follow-up of Q fever patients with regional differences. Therefore the question arose, if and to what extent the serological follow-up rates of Q fever patients differed per LMM catchment area. Are chronic Q fever cases potentially missed due to a lack of proper follow-up? The aim of this study was to investigate the extent to which acute Q fever patients received serological follow-up, identify regional differences and contributing factors and study the differences in knowledge and practices regarding serological follow-up among medical practitioners.

## Methods

### Ethics Statement

According to Dutch legislation, written consent from patients for the use of anonymised information from laboratory databases is not necessary, therefore ethical review was not required.

### Study population and data collection

#### Laboratories of Medical Microbiology (LMMs)

**Figure 1:**
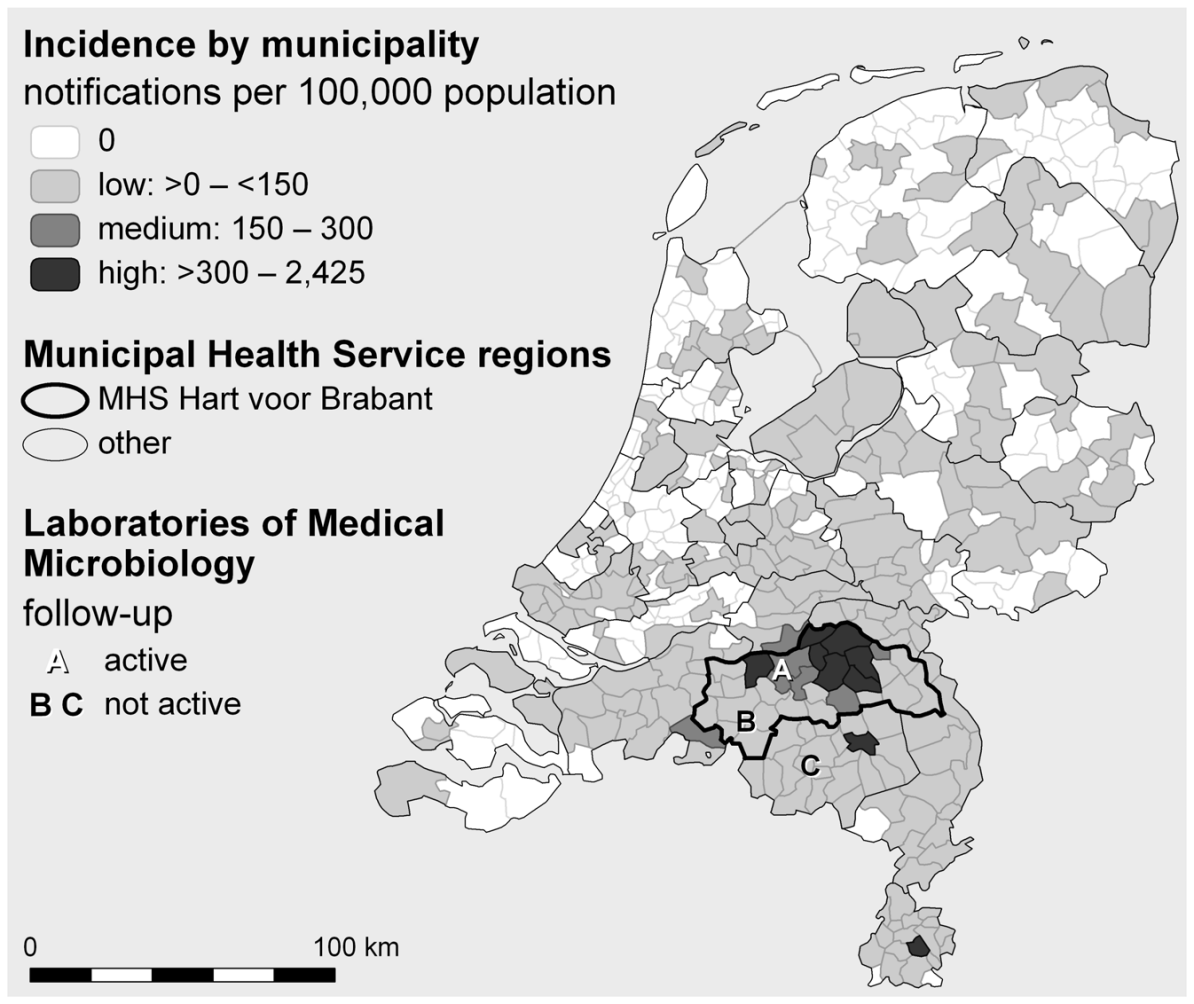
Cumulative Q fever incidence in the Netherlands from 2007 up to and including 2010, marking the Municipal Health Service regions, highlighting the Municipal Health Service region Hart voor Brabant and the Laboratories of Medical Microbiology, A in 's-Hertogenbosch, B in Tilburg, and C in Veldhoven.

Three LMMs (A, B, and C, see Figure 1) performed the majority of Q fever serology in the province of Brabant. LMM-A in 's-Hertogenbosch provided active follow-up by contacting every diagnosed Q fever patient for serological follow-up through an automated system. All patients received an explanatory letter and a laboratory form. The other two LMMs, LMM-B in Tilburg and LMM-C in Veldhoven, performed serological follow-up only upon request of a medical practitioner.

All three LMMs provided anonymous serological datasets from all patients that were diagnosed with acute Q fever between January 2007 and December 2009. Follow-up samples up to 15 months after diagnosis of Q fever were analysed for timing and frequency. Samples that were taken within 60 days of diagnosis were not considered as follow-up samples. Follow-up periods were divided into 60–135 days (2–4.5 months), 136–255 days (4.5–8.5 months) and 256–450 days (8.5–15 months) in order to include the 3-, 6-, and 12-month follow-up, respectively. The 9-month follow-up started in 2010; therefore these data are not presented as a separate follow-up moment in this study but are included in the follow-up period 256–450 days (8.5–15 months). Patients that were present in the dataset of more than one LMM were only included once by checking gender, date of birth and the postal code. These patients were then allocated to the LMM that requested the Q fever serology. We conducted semi-structured interviews with the head medical microbiologist of each laboratory regarding perceived role and responsibility of serological follow-up of Q fever patients.

### Information from general practitioners and medical specialists

In December 2011, questionnaires were posted to all 240 general practices (with 501 general practitioners) and all internists (N = 42), cardiologists (N = 46), and pulmonologists (N = 24) from all hospitals (N = 6) in the MHS region Hart voor Brabant (MHS HvB), the epicentre of the Q fever outbreaks (see Figure 1). We used the term medical practitioners to refer to both general practitioners (GPs) and medical specialists. Non-responders received a reminder after two and four weeks. Reminders were not sent to GPs when one out of three GPs from the medical practice responded.

The questions posed were; work location (postal code), LMM used, the number of Q fever patients treated and the knowledge and practices regarding serological follow-up of Q fever patients. Practice questions included informing the patient about the importance of serological follow-up (never/sometimes/often/always); requesting Q fever follow-up serology for patients (never/sometimes/often/always); and differentiating between high- and low-risk patient groups when offering follow-up (never/sometimes/often/always). Never and sometimes were regarded as inadequate practice. Knowledge questions (multiple-choice) focused on identification of high-risk groups for developing chronic Q fever i.e. “people with valvulopathy, vascular prosthesis/abnormalities, pregnant women, and the immunocompromised”. The possibility to add another perceived risk group was offered as an open question. The same method was used for the follow-up, timing and differences in follow-up between high- and low-risk group patients. Not being able to identify three high-risk groups, and making no distinction in frequency or timing of serological follow-up between high- and low-risk groups were regarded as incorrect answers.

Medical practitioners were divided in groups with zero, few (≤10) and many (>10) Q fever patients and the Q fever incidence area where they worked. These Q fever incidence areas were based on the cumulative Q fever notification data from 2007 up to December 2010 in the area of the MHS HvB and were defined as low (<150 cases per 100,000 residents), medium (150–300/100,000) and high (>300 up to 2,425/100,000) (see Figure 1).

### Data analysis

All data were analysed using SPSS (v. 19) 2010. Proportions were compared with the Mantel–Haenzel chi square and Fishers exact test. P-values were based on two-tailed tests, defining P<0.05 as significant.

## Results

### Laboratories of Medical Microbiology

We received serological datasets of 3,198 patients diagnosed by three LMMS between 2007 and 2009 with serology indicative of acute Q fever (Figure 1). The difference in percentage of patients without serological follow-up within 15 months of diagnosis, differed greatly between LMMs with an active or passive follow-up approach (Table 1); 5% (120/2,346) versus 74% (634/852) respectively (OR 54, 95% CI 43–67). The percentage of patients that did not receive serological follow-up was comparable for the two LMMs without active follow-up (74%). Overall, 24% (754/3,198) of Q fever patients did not receive any follow-up.

**Table 1:** Diagnosis and serological follow-up up to 15 months (450 days) after diagnosis of Q fever for three Laboratories of Medical Microbiology (LMM).

	Provision follow-up service and location LMM			
	Yes	No	No	Total
	's-Hertogenbosch	Veldhoven	Tilburg	All LMM
	N *(%)*	N *(%)*	N *(%)*	N *(%)*
**Total diagnosis Q-fever**	2,346 *(100)*	527 *(100)*	325 *(100)*	3,198 *(100)*
Diagnosis by				
GP	1,786 *(76.2)*	320 *(60.7)*	91 *(28.0)*	2,197 *(68.7)*
Specialist	536 *(22.8)*	207 *(39.3)*	137 *(42.1)*	880 *(27.5)*
Unspecified	24 *(1.0)*	0 *(0.0)*	97 (*29.8*)	121 *(3.8)*
No follow-up	120 *(5.1)*	392 *(74.4)*	242 *(74.5)*	754 *(23.6)*
Received follow-up in days after diagnosis				
*60–135	2,077 *(88.5)*	67 *(12.7)*	47 *(14.5)*	2,191 *(68.5)*
136–255	2,015 *(85.9)*	57 *(10.8)*	40 *(12.3)*	2,112 *(66.0)*
256–450	1,926 *(82.1)*	61 *(11.6)*	24 *(7.4)*	2,011 *(62.9)*
Follow-up requested by				
GP	NA	86 *(46.5)*	43 *(43.9)*	129 *(45.6)*
Specialist	NA	99 *(53.5)*	55 *(56.1)*	154 *(54.4)*
Total	NA	185 *(100)*	98 *(100)* †	283 *(100)*

* A sample taken within 60 days after diagnosis was not considered as a follow-up sample.

† For 13 samples the applicant was unknown (request by an external laboratory).

NA: not applicable.

During the interviews, one of the heads of an LMM (without a follow-up service) stated that both the medical practitioner and the MHS were responsible for the serological follow-up of Q fever patients. The other two microbiologists perceived this to be a shared responsibility between medical practitioners, patients, and the LMM. The microbiologist in charge of LMM-A, the LMM that provided active follow-up, chose a proactive approach at the beginning of the outbreak. The heads of the two LMMs without active follow-up stated that in their opinion an active recall of patients by an LMM was not an option because they regarded this as interfering with the responsibility of the medical practitioner.

### Response questionnaires and interviews medical practitioners

The response rate of general practices was 70% (167/240), and included 42% (209/501) of GPs. The response rate of specialists was 29% (32/112); highest for pulmonologists 37% (9/24) and internists 33% (14/42), and lowest for cardiologists 15% (6/46). The most frequently mentioned reasons for not participating in the study by non-responders who gave reasons (N = 70) were no Q fever patients (38%) or time constraints (25%).

### Knowledge and behaviour of medical practitioners regarding serological follow-up

Although 80% (162/203) of all medical practitioners with Q fever patients reported informing patients of the importance of serological follow-up, 33% (67/203) stated never to request follow-up. Information on knowledge and practice questions for medical practitioners with Q fever patients that do (sometimes/often/always) offer follow-up is provided in table 2. Outcomes were comparable for different incidence areas and type of medical practitioner (GP or medical specialist). Medical practitioners with one to five Q fever patients (mainly found in the low and middle incidence areas) seemed less likely to request serological follow-up, as 47% (27/58) stated never. There was no significant difference compared to those with more patients. Overall, there was no difference in reported practice of requesting follow-up serology between GPs in an area with or without an automatic recall-system (Table 3). GPs with many patients (>10) and working in the catchment area of a LMM without active follow-up requested follow-up significantly more often than those with few patients (≤10).

**Table 2:** Answers to knowledge and practice questions of medical practitioners (MPs) comparing those with few (≤10) and many (>10) Q fever patients.

	Number* of Q fever patients per medical practitioner						
	>10		≤10		Total		
	Answered Yes	Total MPs	Answered Yes	Total MPs	Answered Yes	Total MPs	OR (95% CI)
Knowledge questions	N (%)	N *(100%)*	N (%)	*N (100%)*	N (%)	N *(100%)*	
Makes distinction of risk groups	35 *(50.7)*	69 *(100)*	33 *(53.2)*	62 *(100)*	68 *(51.9)*	131 *(100)*	0.9 (0.4–1.8)
Identifies all high-risk groups	28 *(41.1)*	68 *(100)*	16 *(24.6)*	65 *(100)*	44 *(33.1)*	133 *(100)*	2.1 (1.0–4.5)
**Practice questions**							
Discusses importance of follow-up with patient	66 *(94.2)*	70 *(100)*	55 *(82.1)*	67 *(100)*	121 *(88.3)*	137 *(100)*	3.6 (1.1–11.8)
Requests follow-up Q fever patients without distinction of risk groups	32 *(45.7)*	70 *(100)*	31 *(49.2)*	63 *(100)*	63 *(47.4)*	133 *(100)*	0.8 (0.4–1.7)
Requests serology at least once for low-risk groups	34 *(52.2)*	65 *(100)*	36 *(59.0)*	61 *(100)*	65 *(51.5)*	126 *(100)*	0.7 (0.4–1.5)
Requests serology at least three times for high-risk groups	34 *(57.6)*	59 *(100)*	18 *(30.5)*	59 *(100)*	52 *(44.1)*	118 *(100)*	3.3 (1.4–5.0)

* Excluded are medical practitioners without Q fever patients (n = 30), those who never request serological follow-up (n = 70) or gave not applicable (NA) answers.

**Table 3:** Regional differences in reported serological follow-up practices by GPs in regions with a Laboratory of Medical Microbiology (LMM) with or without an automatic follow-up system.

	Number of GPs by LMM region							
	LMM with automatic follow up; GPs N = *123 (100%)*				LMM without automatic follow-up GPs; N = *47 (100%)*			
Frequency serology request GP	Few patients ≤10	Many patients >10	Total	OR (95% CI)	Few patients ≤10	Many patients >10	Total	OR (95% CI)
	N *(%)*	N *(%)*	N *(%)*	0.6 (0.2–1.2)	N *(%)*	N (%)	N (%)	4.8 (1.1–22.1)
Mostly/always	23 *(44.2)*	22 *(30.9)*	45 *(36.6)*		12 *(32.4)*	7 *(70.0)*	19 *(40.4)*	
Sometimes/never	29 *(55.8)*	49 *(69.1)*	78 *(63.4)*		25 *(67.6)*	3 *(30.0)*	28 *(59.6)*	
Total	52 *(100)*	71 *(100)*	123*(100)*		37 *(100)*	10 *(100)*	47 *(100)*	

Municipalities in the service area of a LMM with follow-up: Heusden, Oss, Maasdonk, Uden, Bernheze, Lith, Landerd, Vught, 's-Hertogenbosch (Den Bosch), Sint Michielsgestel, Veghel, Schijndel, Boekel, Boxtel.

Municipalities in the service area of a LMM without follow-up: Dongen, Waalwijk, Tilburg, Oisterwijk, Gilze Rijen, Loon op Zand, Sint Oedenrode, Cuijk, Boxmeer, Mill en Sint Hubert, Hilvarenbeek, Sint Anthonis, Haaren, Grave.

The ability to differentiate between high- and low-risk patient groups was comparable for GPs and specialists. The knowledge question; “are patients with a heart valve defect a high-risk group for chronic Q fever” was answered ‘yes’ by 88% of GPs and 100% of specialists. For stents and vascular abnormalities this was 85% and 86%, for the immune compromised 85% and 79%, and for pregnant during the initial infection 74% and 61%, respectively. When looking at individual medical practitioners, 67% correctly identified all high-risk groups. When offering serological follow-up, 35% of GPs and 22% of medical specialists never consider the risk category of the patient. Medical practitioners with many (>10) patients scored significantly worse for identification of the correct high-risk groups, discussing the importance of serological follow-up with the patient, and requesting follow-up serology for high-risk groups (Table 2).

Both GPs (63%) and specialists (45%) assumed that the LMM requests follow-up. GPs with few Q fever patients indicated that they were not acquainted with the procedure and referred patients to specialists. The main reason for not requesting serological follow-up, mentioned by GPs with many Q fever patient cases, was the assumption that the LMM or the MHS would take responsibility for this.

## Discussion

### Laboratory follow-up

After diagnosing acute Q fever, serologic follow-up is considered essential for early detection and treatment of chronic Q fever. During the Dutch Q fever outbreak there was no national consensus nor guidelines on serological follow-up of acute Q fever patients. In an attempt to comply with the changing recommendations, LMMs and clinicians improvised. This led to an active recall of patients by one LMM, while in other regions medical practitioners had to organise this follow-up themselves. In this study we analysed the outcome of these two approaches. An active follow-up approach by a LMM led to a much higher follow-up rate compared to follow-up by medical practitioners only (OR 54, 95% CI 43–67). When the responsibility of follow-up lies with medical practitioners, the outcome is poor. Overall, 1,187 (37%) patients received incomplete or no (24%; N = 754) follow-up. Ideally, the percentage of chronic Q fever cases found in the group of patients that did receive follow-up would be known, based on the conversion rate to chronic Q fever. However, the diagnosis of chronic Q fever is a combination of; an IgG phase I antibody titre against *C. burnetii* of ≥1∶1,024 in immunofluorescence assay in a follow-up sample [7], the detection of *C burnetii* DNA in blood or tissue, clinical findings, the presence of risk factors, and diagnostic imaging techniques. This additional information was unavailable. Chronic Q fever is not notifiable and therefore we lacked accurate data on the occurrence of chronic Q fever. We were unable to retrieve accurate data on chronic Q fever from patients that were lost to follow-up, as patient's personal details were removed from the LMM database for reasons of anonymity. We do however know that up to the beginning of 2013 a total of 3% (71/2226) of patients of the LMM that provided active follow-up service had an antibody response (IgG phase I) suspect for a probable, possible or proven chronic Q fever (personal communication, unpublished data Nicole HM Renders, Medical Microbiologist). However, new chronic cases are still being identified, as the average incubation period of chronic Q fever may be long and definitive identification and characterization of chronic Q fever patients is complicated. Based on an estimated 1–5% conversion rate to chronic Q fever, we calculate that approximately 12 to 59 (1–5% of 1,187 patients without or with incomplete follow-up) chronic Q fever patients might have been missed because of inadequate follow-up. Now that it is known that this many traceable patients received no, or improper follow-up, the discussion arises whether offering serological testing years after the initial infection would be beneficial to patients. At the same time the current screening recommendations [14] are questioned. What percentage of chronic Q fever might we expect to find per risk category and how should these categories be defined? Should all 1,187 Q fever patients need to be recalled or only a selection of high-risk patients? What percentage of chronic Q fever patients diagnosed several years after acute Q fever would justify such a recall? Should one incorporate a time limit for follow-up for patients after an acute infection that do not belong to a risk category? Other important issues are the cut-off value of the immunofluorescence assay, and the duration and frequency of follow-up. In the Netherlands, several follow-up studies are currently being conducted that may answer some of these questions.

One would assume that patient compliance is the same regardless of the system. However, computer generated systems are known to improve patient compliance [15] and a diagnosis of acute Q fever made by a laboratory with an active recall-system provides the best guarantee for receiving follow-up. The downsides of such a system are the unnecessary exposure of patients to blood tests and the overburdening of laboratory facilities. To prevent overburdening, the LMM needs clinical information from the medical practitioner to distinguish between acute and old infections [16] and risk categories, but this information is often not provided.

### Response rate, knowledge and practices of medical practitioners

The response rate of the general practices was good (70%), and we consider our sample to be representative for GPs in the MHS region as the proportions of responding practices were comparable for the different incidence areas. We used the number of Q fever patients per GP rather than incidence area because all GPs stated the number of Q fever patients.

Approximately half of the medical practitioners lacked knowledge on high-risk groups, distinction between low- and high-risk patients, and the need to request serological follow-up for all acute Q fever patients. A high proportion of medical practitioners (88%) reported that they discussed the importance of serological follow-up with the patient but it might be that an expected correct answer was given [17].

Barriers to behavioural change by GPs' and specialists' relate to knowledge, attitude and external factors [18], [19]. Although many different parties play a role in serological follow-up, correct information and knowledge [18] is the first step to compliance. During the outbreak, the MHS HvB regularly advised medical practitioners to contact a microbiologist for specific advice on follow-up and dispersed general information on the importance of follow-up in update letters and in every notification report letter (following the notification of a Q fever patient). LMM-A and LMM-C mentioned the required serological follow-up on each Q fever positive laboratory report while LMM-B discussed this with the medical practitioner by telephone. The lack of knowledge amongst medical practitioners may be due to a combination of changing recommendations on Q fever follow-up [9], [12]–[14] combined with a lack of national guidelines (to this date) and general information overload [20].

### Conclusion and recommendations

The serological follow-up of Q fever patients poses logistical challenges. Our results clearly indicate that a LMM based follow-up system with active patient approach achieves high patient compliance compared with systems that rely on referral by medical practitioners. Also, the current registration systems of medical practitioners are not suited to follow-up Q fever patients. Medical practitioners hold others, including the patient, responsible for follow-up and often lack knowledge on the indication for and implementation of serological follow-up of Q fever. A lesson learned from this outbreak, is that recommendations on best practices regarding the serological follow-up of acute Q fever patients should be translated into practical guidelines for medical practitioners early on during an outbreak. The recommendation on serological follow-up should also be incorporated in patient information leaflets. Recalling selected high risk patients that received incomplete or no serological follow-up should be considered. Additional information, on conversion to chronic Q fever per patient category in time, is needed in order to decide which patient groups should be recalled and up to what time after initial infection. Organising such a recall needs to be a joint action by medical practitioners, the LMM, the Q fever patient association and the MHS.
